# Geranylgeranyl diphosphate synthase inhibition induces apoptosis that is dependent upon GGPP depletion, ERK phosphorylation and caspase activation

**DOI:** 10.1038/cddis.2017.101

**Published:** 2017-03-16

**Authors:** Sherry S Agabiti, Jin Li, Andrew J Wiemer

**Affiliations:** 1 Department of Pharmaceutical Sciences, University of Connecticut, School of Pharmacy, Storrs, CT, USA; 2 Institute for Systems Genomics, University of Connecticut, Storrs, CT, USA

## Abstract

Bisphosphonates are diphosphate analogs that inhibit the intermediate enzymes of the mevalonate pathway. Here, we compared the effects of a farnesyl diphosphate synthase inhibitor, zoledronate, and a geranylgeranyl diphosphate synthase (GGDPS) inhibitor, digeranyl bisphosphonate (DGBP), on lymphocytic leukemia cell proliferation and apoptosis. Both zoledronate and DGBP inhibited proliferation with DGBP doing so more potently. DGBP was markedly less toxic than zoledronate toward the viability of healthy human peripheral blood mononuclear cells. Addition of GGPP, but not farnesyl diphosphate (FPP), prevented the anti-proliferative effects of DGBP. Both GGPP and FPP partially rescued the effects of zoledronate. Co-treatment with DGBP and zoledronate was antagonistic. To further assess the effects of the bisphosphonates, we analyzed annexin V and propidium iodide staining via flow cytometry and found that DGBP induced apoptosis more potently than zoledronate. Western blots show that DGBP treatment altered expression and membrane affinity of some but not all geranylgeranylated small GTPases, activated caspases and increased ERK phosphorylation. Importantly, the anti-proliferative effects of DGBP were blocked by treatment with a caspase inhibitor and by treatment with a MEK inhibitor. Together, our findings indicate that DGBP is a more potent and selective compound than zoledronate in inducing apoptosis mediated through pathways that include caspases and MEK/ERK. These findings support the further development of GGDPS inhibitors as anticancer therapeutics.

Bisphosphonates are used widely for treatment of osteoporosis and other indications related to bone and calcium metabolism.^1, 2, 3^ These compounds are structural analogs of diphosphates that are resistant to metabolism because they contain a carbon atom in place of the connecting oxygen atom normally found in the diphosphate.^2, 4^ The bisphosphonate structure is critical for binding to the active sites of pharmacological targets including the enzyme farnesyl diphosphate synthase (FDPS).^5, 6^ At the same time, the bisphosphonate structure impacts the pharmacokinetics of these drugs as it has a strong affinity for binding to calcium, thus promoting bone distribution.^7^ These compounds primarily function by inhibiting cellular functions in the bone microenvironment. This is especially important for osteoporosis therapy because bisphosphonates can reduce osteoclast-mediated bone resorption and ultimately strengthen bone density.^3, 8^

As a result of its activity in the bone microenvironment, the third generation bisphosphonate zoledronate also has become useful for treatment of metastatic bone disease associated with solid tumors,^9, 10, 11, 12^ as well as multiple myeloma.^13, 14, 15, 16, 17^ It is thought that zoledronate functions to reduce the cellular intermediates of isoprenoid biosynthesis including farnesyl diphosphate (FPP) and geranylgeranyl diphosphate (GGPP), which are required for cell proliferation (Figure 1).^18, 19^ This disrupts protein geranylgeranylation, a process often required for malignant cell growth.^20, 21, 22^ However, the mechanisms by which depletion of isoprenoids in transformed cells inhibits proliferation remain unclear. In addition, the possibility remains that zoledronate or other bisphosphonates may also be used for other malignancies, which have bone complications, such as acute T lymphocytic leukemia.^23, 24, 25, 26, 27, 28^

Bisphosphonates may ultimately be beneficial for leukemia therapy because leukemia patients frequently experience bone pain because of accumulation of the leukemia cells in the bone and joints.^28^ In addition, a substantial number of patients experience hypercalcemia, in particular those with leukemias derived from T cells.^29^ Therefore, bisphosphonates may offer two disease-modifying mechanisms to T-cell leukemia – direct inhibition of leukemia cell proliferation that results from their inhibition of isoprenoid biosynthesis^28^ and relief from hypercalcemia that results from their binding to calcium ions.^7^

Although the clinically used bisphosphonates inhibit the enzyme FDPS,^30, 31, 32, 33, 34^ we have recently explored a new class of bisphosphonates including digeranyl bisphosphonate (DGBP; Figure 1), which target the subsequent enzyme in the mevalonate pathway,^35^ geranylgeranyl diphosphate synthase (GGDPS).^36, 37, 38^ The downstream molecular target affords the opportunity to retain the anti-proliferative characteristics of zoledronate, which can result from depletion of GGPP while reducing potential side effects that may occur from depletion of FPP. Here, we evaluate the efficacy by which these two classes of bisphosphonates induce cell death in T lymphocytic leukemia and provide insights into the mechanisms through which depletion of isoprenoids leads to leukemia cell death.

## Results

### DGBP inhibits proliferation of lymphocytic leukemia cell lines more potently than zoledronate

**Figure 2:**
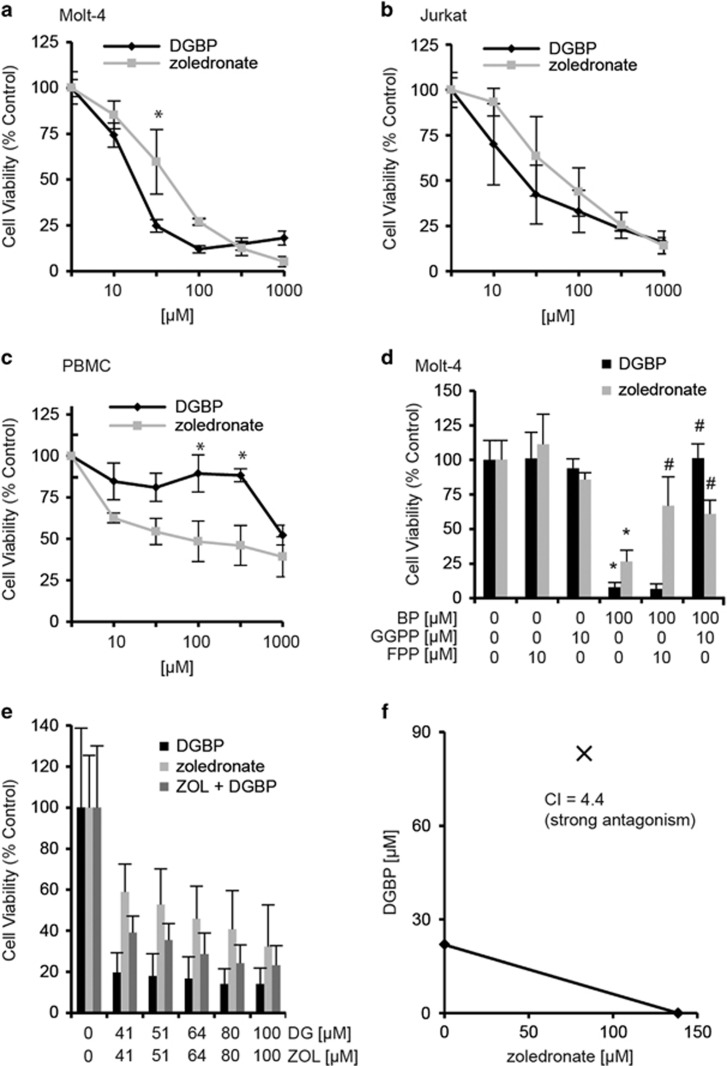
DGBP and zoledronate inhibit proliferation of malignant T lymphocytic cells through depletion of GGPP. (**a**-**c**) Dose-response curves for Molt-4 and Jurkat cells, and PBMC, treated with either DGBP or zoledronate. Cells were incubated for 72 h with compound. *Indicates significant difference between the two compounds at the indicated drug concentration. (**d**) Growth inhibition caused by DGBP is rescued by co-treatment with GGPP, and not rescued by FPP, whereas zoledronate is partially rescued by co-treatment with either GGPP or FPP. (**e**) Proliferation of Molt-4 cells with DGBP in combination with zoledronate. (**f**) Isobologram of DGBP with zoledronate. *Indicates significant difference with respect to untreated control. ^#^Denotes significant difference from treatment with bisphosphonate alone. Statistical significance determined by ANOVA with *P*<0.05 as significant with Tukey's post-hoc analysis. *n*=3 for all proliferation experiments

We first compared the effects of two nanomolar inhibitors: zoledronate (a potent clinical inhibitor of FDPS)^39^ and DGBP (a specific experimental inhibitor of GGDPS)^37^ on the proliferation of T-cell leukemia lines. Treatment of either Molt-4 or Jurkat cells with varied concentrations of either compound for 72 h dose-dependently inhibited cell proliferation (Figures 2a and b). In both the Molt-4 and Jurkat cell lines, the anti-proliferative effect of DGBP was stronger than that of zoledronate (half-maximal inhibitory concentration (IC_50_) of DGBP=15 *μ*M in Molt-4 cells and 30 *μ*M in Jurkat cells, IC_50_ of zoledronate=52 *μ*M in Molt-4 cells and 81 *μ*M in Jurkat cells) (Table 1). Both compounds were more potent against proliferation of these lymphocytic cell lines compared with myeloid leukemias, for which concentrations above 100 *μ*M were required.^40^ This surprising effect suggests that lymphocytic leukemias are more sensitive to isoprenoid depletion relative to myeloid leukemias and that inhibition of GGDPS is more anti-proliferative than inhibition of FDPS.

### Zoledronate but not DGBP decreases viability of primary PBMCs

To determine whether the compounds have preferential effects on viability of malignant *versus* normal cells, we tested zoledronate and DGBP for toxicity against primary human peripheral blood mononuclear cells (PBMCs). In contrast to the pattern observed in the leukemia cell lines, zoledronate exhibited a stronger cytotoxic effect against the primary cells than DGBP (Figure 2c). The 72-h IC_50_ of zoledronate against PBMCs was 63 *μ*M, whereas the IC_50_ for DGBP was 1200 *μ*M. A therapeutic index was determined for each drug by calculating the ratio of the IC_50_ values in the leukemia cell lines *versus* PBMCs (Table 1), which demonstrates that in contrast to zoledronate, which offers no selectivity, DGBP is much more selective (80- to 40-fold) against actively proliferating leukemia cells over resting primary blood cells. This finding also suggests that inhibition of FDPS by zoledronate results in cytotoxic effects in addition to the anti-proliferative effects caused by its depletion of GGPP.

### GGPP is required for proliferation

To confirm target engagement in these cells, we performed add-back experiments by treatment of the cells with either of the two bisphosphonates for 72 h in the presence or absence of exogenous GGPP or FPP. As expected, co-treatment with GGPP fully prevented the anti-proliferative effect of the GGDPS inhibitor, DGBP, whereas FPP did not. In these cells, treatment with GGPP resulted in a partial rescue of the zoledronate anti-proliferative effect, as did FPP (Figure 2d). Thus, zoledronate exerts its anti-proliferative effects in part via depletion of GGPP.

### The combination of DGBP and zoledronate is antagonistic in Molt-4 cells

To determine if the combination of DGBP and zoledronate more strongly inhibits proliferation of Molt-4 cells, we treated cells for 72 h with varied doses of the two compounds in a constant ratio determined by their respective potencies. Previously, in the K562 myeloid leukemia line, we observed weak synergy between the two compounds.^41^ However, in the Molt-4 T-cell leukemia line, we observed strong antagonism with this combination (Figures 2e and f). Here, the combination index values at the experimental concentrations ranged from 2 to 5 (Supplementary Table S1). Therefore, the use of dual inhibitors of the FDPS and GGDPS enzymes is not likely to result in enhanced inhibition of proliferation with respect to T-cell leukemias.

### DGBP treatment alters protein expression and membrane association of RhoA, Rac and Rap1 but not Cdc42

**Figure 3:**
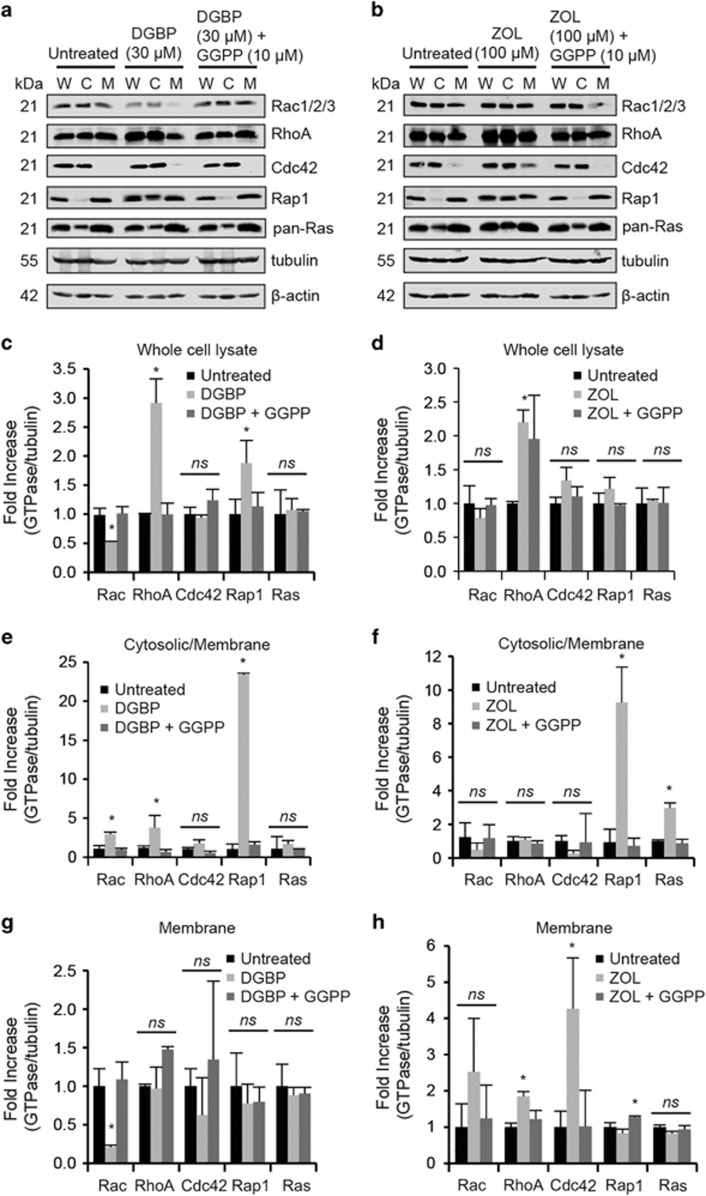
DGBP alters membrane levels of Rac but not RhoA or Cdc42. (**a**) Western blot of Rac1/2/3, RhoA, Cdc42, Rap1 and pan-Ras in Molt-4 cells treated with DGBP. ‘W' represents whole-cell lysate, ‘C' represents the cytosolic fraction and ‘M' represents the membrane fraction. (**b**) Western blot of RhoA, Rac1/2/3, Cdc42, Rap1 and pan-Ras in Molt-4 cells treated with zoledronate and co-incubated with GGPP. (**c**) Quantification of whole-cell lysate fractions of small GTPases with DGBP. (**d**) Whole-cell lysate fractions of small GTPases with zoledronate. (**e**) Cytosolic fractions *versus* membrane fractions of cells treated with DGBP. (**f**) Ratio of cytosolic fractions: membrane fractions of cells treated with zoledronate. (**g**) Quantification of membrane fractions of Molt-4 cells treated with DGBP and GGPP on left and (**h**) zoledronate and GGPP on right. All data represented as mean±S.D., *n*=3, *P<0.05 by ANOVA with Tukey's post-hoc analysis

We examined the effect of DGBP on levels and membrane localization of a panel of geranylgeranylated proteins including RhoA, total Rac, Cdc42 and Rap1 (Figures 3a and b). Ras, expected to be predominately farnesylated, was used as a control. Treatment with DGBP increased protein expression of RhoA and Rap1 and decreased expression of total Rac (Figure 3c). These changes in protein expression caused by DGBP were prevented by co-incubation with GGPP. DGBP did not alter Cdc42 and Ras protein expression (Figure 3c). Treatment with zoledronate increased the expression of RhoA, which could not be rescued by GGPP co-incubation (Figure 3d). Zoledronate treatment did not appear to alter levels of Rac and Rap1.

As expected, a significant fraction of RhoA, Rac, Rap1 and Ras was membrane associated (Figures 3a and b). Cdc42 was primarily cytosolic. DGBP treatment increased the ratio of cytoplasmic to membrane-associated RhoA, Rac and Rap1, but not Ras (Figure 3e). In DGBP-treated cells, this change in RhoA and Rap1 resulted from increased total protein, which accumulated in the cytosolic fraction. However, the change in Rac localization was driven by decreased total protein levels, which also were decreased in the membrane fraction (Figure 3g). The changes in Rac, RhoA and Rap1 localization induced by DGBP treatment were restored upon co-incubation with GGPP.

Treatment with zoledronate altered Rap1 and Ras cytosolic to membrane ratios (Figure 3f). Although zoledronate reduced GGPP levels as evidenced by its effects on Rap1, zoledronate did not alter the cytosolic to membrane ratios of Rac or RhoA. Interestingly, zoledronate caused Cdc42 to be localized in the membrane, whereas DGBP did not have that effect (Figure 3h). Co-incubation with GGPP restored the effect of zoledronate on Rap1 and Ras. Taken together, treatment with DGBP broadly affects the expression and localization of geranylgeranylated proteins and does so with a different pattern of activity than zoledronate.

### DGBP-induced inhibition of proliferation is not mediated by geranylgeranylated RhoA

**Figure 4:**
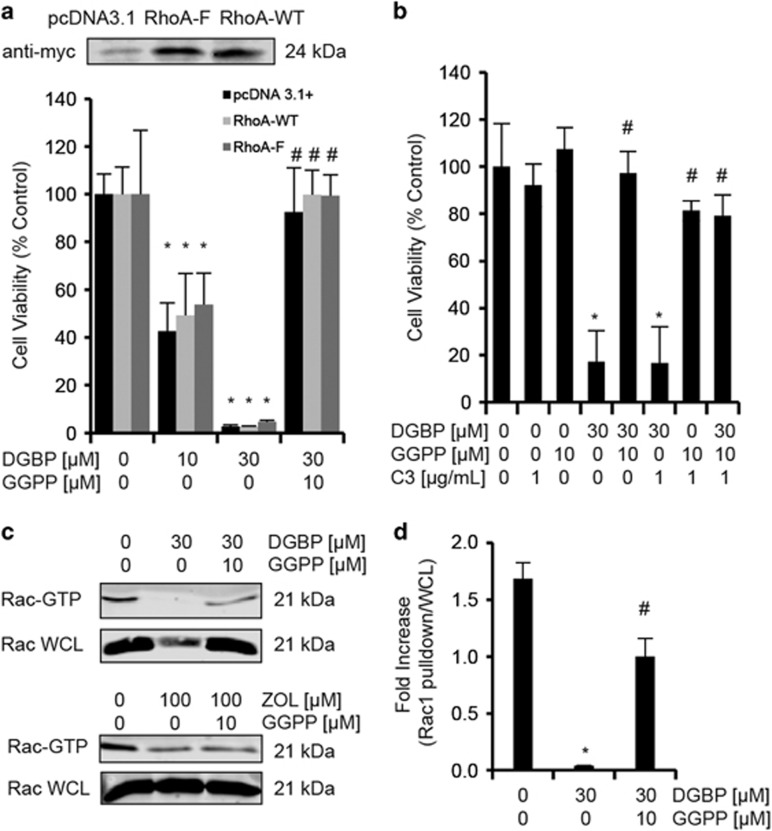
Neither Rho nor Rac is responsible for mediating the anti-proliferative effect of DGBP. (**a**) Farnesylated RhoA does not bypass the effect of DGBP. Seventy-two-hour proliferation of transfected Molt-4 cells. pcDNA3.1 represents the empty vector. Cells were treated with DGBP alone and DGBP with GGPP. (**b**) Cells were treated with C3 transferase alone and in combination with DGBP and GGPP for 72 h. Proliferation is determined by CellQB assay. *Indicates significance with respect to untreated control. ^#^Denotes significant difference from treatment with bisphosphonate alone. (**c**) Western blot analysis of activated Rac1 pull-down. ‘WCL' represents the whole-cell lysate. (**d**) Quantification of western blot analyses of Rac1 (right). Both quantifications were compared with whole-cell levels of GTPase. All data represented as mean±S.D. for *n*=3

This pattern of GTPase regulation in response to DGBP treatment initially led us to hypothesize that the anti-proliferative effects of DGBP were mediated by its effects on RhoA. We asked whether or not overexpression of RhoA could prevent the anti-proliferative effect of DGBP (Figure 4a). We expressed exogenous WT-RhoA and found it was unable to restore the anti-proliferative effect of DGBP. As the plasmid WT-RhoA would also be geranylgeranylated and blocked by DGBP, we also expressed a mutated RhoA in which the CAAX motif^42^ was replaced with one signaling farnesylation (CVLS). Again, farnesylated RhoA was unable to block the anti-proliferative effects of DGBP, as DGBP decreased proliferation of cells transfected with RhoA-CVLS, as well as those transfected with the pcDNA3.1+ (empty vector) (Figure 4a).

As it has been reported that active cytosolic RhoA can mediate effects of isoprenoid depletion in some systems,^43^ we tested whether inhibition of Rho affects DGBP activity. Treatment with a cell permeable C3 transferase, which inhibits Rho activity and has low affinity for Cdc42 and Rac, had no effect on the proliferation in the presence of DGBP or GGPP (Figure 4b). Taken together, this implies that a Rho GTPase, including RhoA, is not the primary GTPase involved in the anti-proliferative effects of DGBP in this model.

### DGBP decreases levels of active Rac

As we observed that DGBP treatment strongly decreased levels of membrane-associated Rac (Figure 3), we assessed how DGBP treatment affects Rac activation as monitored by its GTP binding. We pulled down GTP-bound Rac1 using PAK1-conjugated GST beads and determined levels of active GTP-bound Rac by western blot. GTP-bound Rac1 decreased with DGBP treatment (Figures 4c and d), and was restored to control levels with GGPP co-incubation, suggesting that GGPP depletion decreases the activity of Rac1. We found that NSC 23766, a Rac1 inhibitor, has similar effects on the proliferation as DGBP does (Supplementary Figure 1). Taken together, DGBP treatment reduces Rac1 expression, localization and GTP binding.

### DGBP induces apoptosis more potently than zoledronate

**Figure 5:**
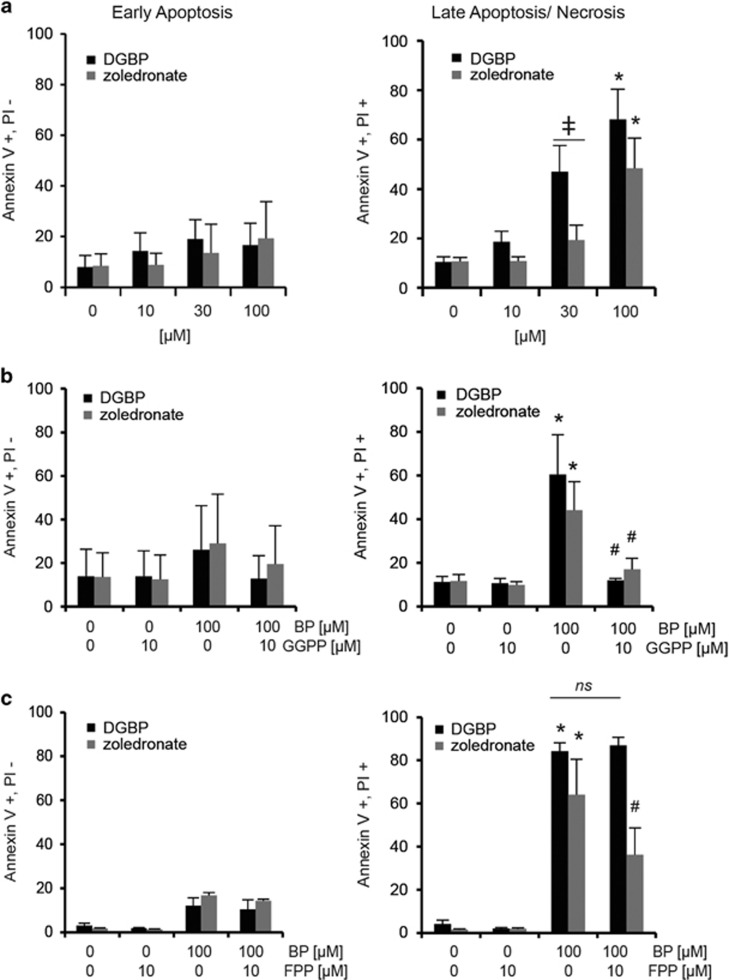
DGBP and zoledronate induce late apoptosis through depletion of GGPP. (**a**) Annexin V/PI staining of Molt-4 cells as determined by flow cytometry. Dose response of DGBP and zoledronate. Left: early apoptosis, right: late apoptosis. (**b**) Bisphosphonates with co-incubation of GGPP. Cells were incubated for 72 h with various concentrations of bisphosphonate. Left: early apoptosis, right: late apoptosis. (**c**) Bisphosphonates with co-treatment of FPP. Left: early apoptosis, right: late apoptosis. All data represented as mean±S.D., *n*=3, *indicates significant difference with respect to untreated control conditions; ^#^indicates significant difference with respect to bisphosphonate-treated conditions. ^‡^Denotes significant difference between indicated populations. Statistical significance determined by ANOVA with *P*<0.05 and with Tukey's post-hoc analysis

To determine whether the anti-proliferative effects of these bisphosphonates resulted from increased apoptosis, we analyzed annexin V and propidium iodide (PI) staining. Cells were treated with DGBP or zoledronate for 72 h at varied concentrations. Both compounds increased the percentage of cells in late apoptosis (annexin V+/PI+) but not early apoptosis (annexin V+/PI−) (Figure 5a). Consistent with the proliferation experiments, DGBP was also more potent than zoledronate for induction of apoptosis, with a statistically significant difference observed when cells were treated with 30 *μ*M of either agent (Figure 5a). In these experiments, co-incubation with GGPP rescued the ability of both bisphosphonates to induce late apoptosis (Figure 5b). FPP did not rescue the effect of DGBP, but did partially rescue zoledronate (Figure 5c).

**Figure 6:**
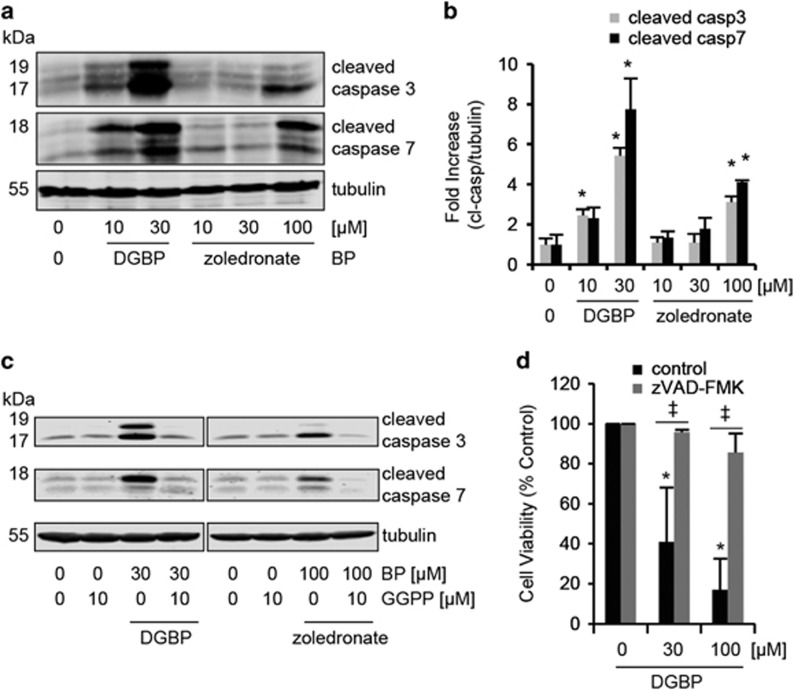
DGBP and zoledronate-mediated apoptosis is through caspase 3 and 7 cleavage. (**a**) Western blot of cleaved caspase 3/7 in Molt-4 cells with bisphosphate treatment. (**b**) Caspase 3/7 cleavage is greater with DGBP than zoledronate. (**c**) Caspase cleavage is rescued by co-incubation with GGPP. (**d**) Proliferation of Molt-4 cells in presence of different concentrations of DGBP and 100 *μ*M zVAD-FMK (pan-caspase inhibitor). In **b** and **d**, all data represented as mean±S.D., *n*=3, *indicates significant difference with respect to untreated control conditions. ^‡^Denotes significant difference between indicated populations. Statistical significance determined by ANOVA with *P*<0.05 and with Tukey's post-hoc analysis. Data represented as mean±S.D. and *n*=3

In addition, induction of apoptosis was examined by western blot analysis for the appearance of caspase 3 and caspase 7 cleavage products (Figures 6a and b). DGBP markedly increased both cleaved caspase 3 and caspase 7 at concentrations of 10 and 30 *μ*M. In contrast, zoledronate treatment did not cause cleaving of these caspases at equivalent concentrations. However, zoledronate at 100 *μ*M did induce cleaved caspase, but the magnitude of this effect was less than that of DGBP at significantly lower concentrations. Caspase activation by both bisphosphonates was prevented by co-incubation with GGPP (Figure 6c). Next, we assessed whether the caspase activation was responsible for the anti-proliferative effect of DGBP. To this end, we treated cells with DGBP in the presence or absence of the pan-caspase inhibitor zVAD-FMK (Figure 6d). Again, DGBP dose-dependently inhibited proliferation, which was fully blocked by co-incubation with the caspase inhibitor. Taken together, the mechanism by which DGBP and zoledronate reduce proliferation is by induction of caspase-mediated apoptosis, with DGBP exhibiting greater potency.

### DGBP-induced apoptosis is mediated by ERK activation

**Figure 7:**
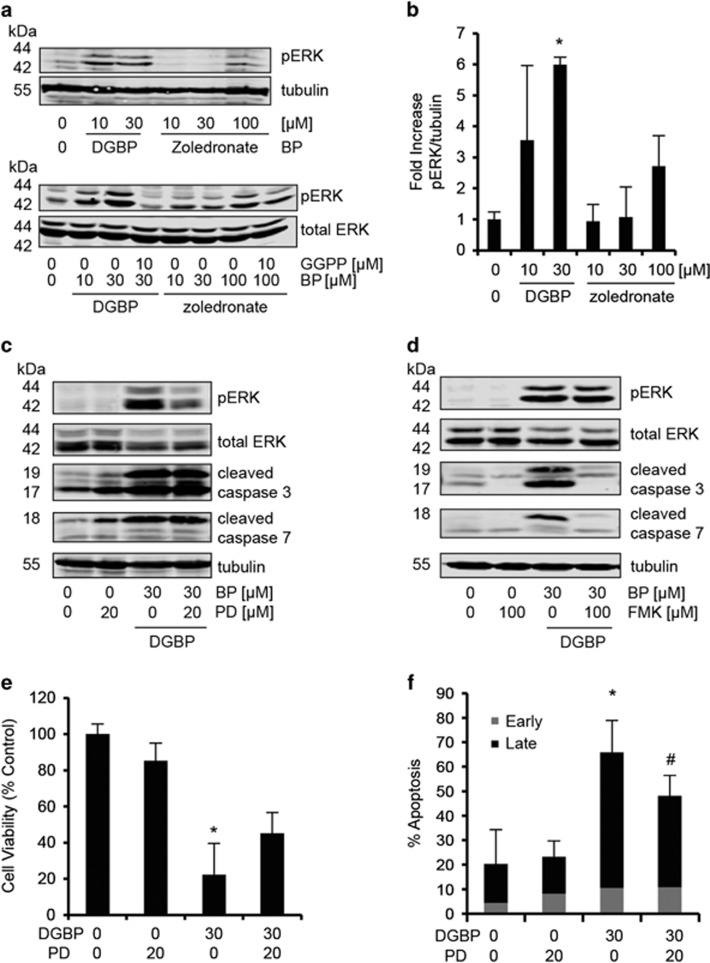
DGBP-mediated apoptosis is partially dependent on ERK. (**a**) Western blot showing ERK phosphorylation in Molt-4 cells treated with bisphosphonates. Data are representative of three independent experiments. (**b**) ERK phosphorylation is dose-dependently increased by DGBP. The effect is greater with DGBP than with zoledronate. (**c**) Western blot analysis of p-ERK, ERK, cleaved caspase 3 and 7 in response to DGBP and PD98059. (**d**) Western blot analysis of p-ERK, ERK, and cleaved caspase 3 and 7 in response to DGBP and zVAD-FMK treatment. (**e**) Molt-4 proliferation with DGBP and PD98059 (MEK inhibitor) treatment via viability assay. (**f**) Molt-4 apoptosis with DGBP and PD98059 treatment via flow cytometry. Annexin V+ and PI− represented by early apoptosis. Annexin V+ and PI+ represented by late apoptosis. All data in **b**, **e** and **f** represented as mean±S.D., *n*=3. *Indicates significant difference with respect to untreated control conditions.^#^Indicates significant difference with respect to bisphosphonate-treated conditions. Statistical analysis done by ANOVA with Tukey's post-hoc analysis with *P*<0.05

As a prior study had suggested that depletion of isoprenoids following treatment with an HMG-CoA reductase inhibitor led to alterations in kinase activity,^44^ we investigated the mechanism by which DGBP induces apoptosis by performing western blot analysis to detect phosphorylation of the mitogen-activated protein kinases ERK, p38 kinase and JNK. We were unable to detect any changes in phosphorylation of p38 or JNK (data not shown), however, treatment with DGBP increased phosphorylated ERK, which was prevented by addition of exogenous GGPP (Figures 7a and b). The increase in ERK phosphorylation was apparent at 10 and 30 *μ*M concentrations of DGBP and 100 *μ*M zoledronate, consistent with concentrations that induced caspase cleavage for both compounds. In addition, co-incubation of GGPP with DGBP decreases levels of phosphorylated ERK (Figure 7a).

We next assessed whether DGBP-induced ERK phosphorylation could be blocked by PD98059, a MEK inhibitor. The induction of ERK phosphorylation by DGBP decreased by co-incubation with PD98059 (Figure 7c), demonstrating that MEK mediates DGBP-induced ERK phosphorylation. At the same time, ERK phosphorylation appears not to be upstream of caspases as the MEK inhibitor did not prevent the effect of DGBP on caspase cleavage (Figure 7c). To further assess the order of activation, we tested whether caspase inhibition would affect ERK phosphorylation (Figure 7d). Again, cells treated with DGBP displayed high levels of cleaved caspase, which was blocked by co-incubation with zVAD-FMK. DGBP-induced ERK phosphorylation was not altered by co-treatment with zVAD-FMK. This finding suggests that MEK activation leading to ERK phosphorylation is not downstream of caspase activation.

We then tested whether ERK phosphorylation was required for inhibition of proliferation and induction of apoptosis by DGBP. We treated cells with DGBP in the presence or absence of PD98059. As a single agent, PD98059 did not affect the cells at concentrations up to 100 *μ*M for 72 h in either the proliferation or apoptosis assays. However, PD98059 was able to partially rescue the anti-proliferative effect (Figure 7e) and the pro-apoptotic effect of DGBP (Figure 7f). Therefore, MEK activity and ERK phosphorylation appears to contribute to DGBP-induced apoptosis.

## Discussion

Here, we have demonstrated that zoledronate and DGBP inhibit proliferation of T-cell leukemia cells via blocking the intermediate enzymes of the mevalonate pathway. This leads to depletion of cellular GGPP, alterations in protein geranylgeranylation and expression, activation of caspases, and pro-apoptotic signaling through ERK. However, these two bisphosphonates, which have different molecular targets, differ in their potency, selectivity and their effects on geranylgeranylated proteins.

DGBP inhibits proliferation and induces apoptosis at lower concentrations than zoledronate, which is consistent with results observed in a prostate cancer model, where DGBP also was more potent.^45^ These results are surprising because zoledronate is approximately 10-fold more potent against purified FDPS than is DGBP against purified GGDPS.^37^ We believe the different pattern in cellular activity is a result of a combination of two factors – (1) higher lipophilicity of DGBP relative to zoledronate, which enhances access to the cellular enzymes and (2) decreased ability of cells to overcome GGDPS *versus* FDPS inhibition.^38^ Although both compounds are active in cells in the micromolar range, bisphosphonates achieve locally high bone concentrations, which enable their effects *in vivo*.^46, 47^

Initially, we hypothesized that increased cellular potency of DGBP relative to zoledronate was due to higher permeability compared with zoledronate. However, if cell permeability were the major issue underlying activity, then one may see a similar pattern of activity in the primary PBMCs. But this pattern is reversed in PBMCs, with zoledronate being more toxic relative to DGBP. This finding argues that the differences in activity between the two compounds results from mechanistic differences caused by inhibition of their respective targets. Indeed, one advantage of targeting GGDPS is that its inhibition cannot be readily overcomed by increased isoprenoid flux.^38^

GGDPS inhibitors have two immediate effects – depletion of the product GGPP and elevation of the reactants isopentenyl diphosphate and FPP. In myeloid leukemia cells, we previously observed that co-incubation with GGPP was unable to mitigate all of the effects of DGBP and that elevated levels of FPP can contribute to the anti-proliferative mechanism.^41^ This differs from the current study in which the effects of DGBP were fully rescued by GGPP. Furthermore, the apoptotic effect of DGBP could not be rescued by FPP. Interestingly, the apoptotic effect of zoledronate was only partially rescued by FPP and was nearly fully rescued by GGPP. Taken together, the apoptotic effects of DGBP and zoledronate in these cells are primarily due to GGPP depletion. As FPP only partially rescued the effects of zoledronate, it is possible that zoledronate may affect GGDPS at high cellular concentrations. In a previous study, zoledronate did not inhibit purified GGDPS up to 10 *μ*M.^37^ We predict that cell permeability is a barrier for entry resulting in lower cellular levels than that are found in the media, but we cannot exclude the ability of zoledronate at 100 *μ*M to inhibit GGDPS, especially given that GGPP rescues its apoptotic effect.

We observed membrane populations of Rac, RhoA and Rap1 but not Cdc42. GGDPS inhibition increased the cytosolic:membrane ratio of all of the membrane-associated geranylgeranylated GTPases examined. Surprisingly, although zoledronate increased the cytosolic:membrane ratio of both Ras and Rap1, it did not significantly alter Rac or RhoA in this manner, in contrast to a prior report that showed zoledronate caused cytoplasmic Rho accumulation.^48^ We found it interesting that Rac and Rho were only altered by DGBP. Although it appears that the effect on Rap1 exceeds the effect on Rac and Rho, there is little Rap1 in the cytosol of untreated cells and nearly no change in the membrane fraction. In fact, of all the proteins examined, only the membrane fraction of Rac in response to DGBP was decreased, whereas the other proteins increased their cytosolic fractions. Although membrane association is not always a prerequisite for activity, it is commonly required for activity and its disruption is desirable.^49, 50, 51^ Ultimately, the localization of many small GTPases was altered with either compound, indicative of engagement with their respective targets. Interestingly, DGBP treatment altered total expression of Rac, RhoA and Rap1, albeit in different ways, with Rac expression decreased and RhoA and Rap1 expression increased. Clearly, different pathways must regulate the expression of these proteins.

Given the effects of DGBP on numerous geranylgeranylated proteins, we found it difficult to assess which specific GTPase, if any, is responsible for inhibition of proliferation. We predicted that RhoA was involved as the bulk of the literature suggests it often mediates proliferation,^52^ and a well-constructed study showed farnesylated RhoA rescued the effect of GGTase I depletion,^22^ but none of the Rho GTPases were involved in the anti-proliferative effects of DGBP. As an alternative, the Rac1 inhibitor caused a decrease in proliferation at a concentration similar to that of DGBP. Although NSC 23766 does not activate ERK (Supplementary Figure S1), Rac may still be involved in DGBP-mediated apoptosis, as others have seen Rac1 is required for proliferation in response to GGTase I overexpression.^53^

How then, does DGBP lead to apoptosis? We hypothesized that MAPK signaling may be affected by changes in protein geranylgeranylation, and found that levels of phosphorylated ERK were altered by DGBP and, to a lesser extent, zoledronate. Therefore, to investigate the ERK pathway as a mechanism of apoptosis, we focused on DGBP, which increased ERK phosphorylation by several fold. This increase in ERK phosphorylation is in contrast to the effect seen in AML as proliferation is blocked by lovastatin and is dependent upon decreased ERK phosphorylation.^54^ There is some precedent for ERK involvement in apoptosis as ERK mediates apoptosis caused by cisplatin.^55^ Although we could not establish a causal relationship between p-ERK and caspase, our data that show DGBP-mediated apoptosis are partially rescued by a MEK inhibitor, suggesting that ERK involvement in apoptosis is late in the stages of apoptosis. Depleting GGPP alters GTPase localization, which cleaves caspases leading to apoptosis and phosphorylates ERK also leading to cell death.

In conclusion, in order to use bisphosphonates to best achieve direct anticancer effects in the bone environment, the general strategy of targeting GGDPS likely compares well with targeting FDPS, which is exemplified by increases in cellular potency and selectivity of DGBP *versus* zoledronate. DGBP works by inhibiting prenylation of some small GTPases, which leads to changes in their expression, caspase cleavage, MEK activation, ERK phosphorylation and apoptosis. Thus, GGDPS inhibitors such as DGBP may produce greater single-agent anti-proliferative effects relative to zoledronate. In addition, future studies should examine whether GGDPS inhibitors or their prodrugs^56^ may be a viable strategy to targeting diseases in which Rac has a role, given the strong reduction in Rac expression, membrane localization and GTP binding seen with DGBP treatment.

## Materials and methods

### Cells and reagents

The T-cell leukemia lines Molt-4 and Jurkat were obtained from ATCC (Manassas, VA, USA). Human PBMCs were purified from whole blood obtained from Research Blood Components (Boston, MA, USA).^57^ Annexin V FITC and PI were obtained from BD Biosciences (Franklin Lakes, NJ, USA) and eBioscience (San Diego, CA, USA). GGPP and FPP were obtained from Cayman Chemical (Ann Arbor, MI, USA). Zoledronate was obtained from Fisher (Rockford, IL, USA). DGBP was a kind gift from Dr. David Wiemer at the University of Iowa, Iowa City, IA, USA. FBS and other tissue culture supplies were obtained from Thermo Fisher (Waltham, MA, USA). CellQuantiBlue was obtained from BioAssay Systems (Hayward, CA, USA). zVAD-FMK was obtained from ApexBio (Houston, TX, USA). PD98059 was obtained from LC Labs (Woburn, MA, USA). C3 transferase was obtained from Cyotoskeleton (Denver, CO, USA). Antibodies to ERK (L34F12) pERK (D13.14.4E), cleaved caspase 3 (5A1E), cleaved caspase 7 (D6H1), RhoA (67B9), Rac1/2/3 (polyclonal #2465) and Cdc42 (11A11) were obtained from Cell Signaling Technology (Danvers, MA, USA). The antibody for beta-actin (Poly6221) was obtained from Biolegend (San Diego, CA, USA). The antibody for Rap1 (EP878) was obtained from Epitomics (Burlingame, CA, USA). The antibody for pan-Ras (C-4) was obtained from Santa Cruz Biotechnology (Santa Cruz, CA, USA). The anti-tubulin (E7) antibody developed by Michael Klymkowsky was obtained from the Developmental Studies Hybridoma Bank at the University of Iowa. pGEXTK-Pak1 70-117 was obtained from Addgene (Cambridge, MA, USA).

### Proliferation/viability assay

Assays were performed as described previously.^58, 59, 60^ Briefly, 10 000 cells per well of Molt-4 or Jurkat cells in log phase growth or 100 000 cells per well of PBMCs were added to 96-well plates in 100 *μ*l in the presence of test compounds and fresh media. Cells were cultured for 72 h. During the last 2 h, cells were labeled with 10 *μ*l of CellQuantiBlue reagent, and were scanned with a Victor PerkinElmer (Waltham, MA, USA) plate reader (ex550/em600).

### Analysis of apoptosis

Annexin V and PI analysis was performed according to the manufacturer protocol (BD Biosciences) as described.^41^ Treated cells were transferred to microcentrifuge tubes, centrifuged at 600 × *g* for 3 min, and then the supernatant was aspirated. Cells were resuspended in 100 *μ*l of binding buffer (10 mM HEPES, 150 mM NaCl, 1 mM MgCl_2_, 5 mM KCl and 1.8 mM CaCl_2_, pH 7.4) then transferred to polystyrene test tubes. Three microliters of FITC annexin V was added, and the cells were incubated for 15 min on ice. Two microliters of 50 *μ*g/ml PI solution (Sigma-Aldrich, St. Louis, MO, USA) was added to the cell suspension, and the suspension was mixed and analyzed using a FACSCalibur (BD Biosciences, Franklin Lakes, NJ, USA).

### Triton X-114 separation

Membrane and cytosolic fractions were purified as described previously with some modifications.^45, 61^ Briefly, cells were resuspended in media at a concentration of 0.75 million cells/ml and cultured for 48 h with indicated concentrations of compounds or solvent controls. Cells were then spun at 600 × *g* for 3 min, washed with PBS, and resuspended in Triton X-114 lysis buffer (20 mM Tris pH 7.5, 150 mM NaCl, 1% Triton X-114) containing freshly added protease and phosphatase inhibitors including leupeptin (1 *μ*g/ml), aprotinin (1 *μ*g/ml), pepstatin (1 *μ*g/ml) and PMSF (200 *μ*M). Lysate was passed through a 27-gauge needle and centrifuged for 15 min at 12 000 × *g* at 4 °C. The resulting supernatant was transferred to a new tube and incubated at 37 °C for 10 min in a water bath. Following incubation, the lysate was centrifuged for 12 000 × *g* for 2 min at room temperature. Aqueous phase (upper) was transferred to a new tube and detergent phase (bottom) was diluted with excess buffer.

### Western blot analysis

Cells were resuspended in fresh media at a concentration of 0.5 million cells/ml. Cells were cultured for 72 h with indicated concentrations of test compounds or solvent controls. Cells were washed once in PBS and resuspended in in lysis buffer (25 mM Tris-HCl pH 7.6, 150 mM NaCl, 1% NP-40, 1% sodium deoxycholate, 0.1% SDS) for 10 min on ice followed by centrifugation for 10 min at 10 000 × *g*. Lysis buffer contained a panel of freshly added protease and phosphatase inhibitors including leupeptin (1 *μ*g/ml), aprotinin (1 *μ*g/ml), PMSF (200 *μ*M), sodium vanadate (200 *μ*M), sodium pyrophosphate (10 *μ*M), sodium fluoride (50 *μ*M) and glycerophosphoric acid (10 *μ*M) (all from Fisher). Proteins were quantified by BCA assay and equivalent masses were loaded onto 10% or 15% SDS-PAGE gels for separation. Proteins were transferred to nitrocellulose membranes and blotted with pERK, ERK, RhoA, Rac1, Cdc42, Rap1, pan-Ras, cleaved caspase 3 or cleaved caspase 7 antibodies. Proteins were visualized using a Licor (Lincoln, NE, USA) Odyssey. Alexa-Fluor 680 goat-anti-mouse IgG and IRDye 800CW goat-anti-rabbit IgG were used for detection. Tubulin or beta-actin were used as a loading controls.

### Rac1 pull-down assay

*E. coli* (BL21) were transformed with pGEXTK-Pak1 70-117. In all, 50 ml cultures in LB-AMP were grown for 14 h. The culture was transferred to a flask of 750 ml of LB broth and treated with IPTG (1 mM) for 5 h. Cells were pelleted by centrifugation and resuspended in 25 ml of lysis buffer (50 mM sodium phosphate, 300 mM NaCl, pH 8.0 with PMSF, aproptinin, leupeptin and pepstatin). The lysate was passed through a French press twice. The lysate was then allowed to bind to glutathione agarose beads overnight at 4 °C (Thermo Fisher). GST beads were washed with equilibration buffer (50 mM Tris, 150 mM NaCl, pH 8.0) and stored at −80 ºC. Molt-4 cells were lysed according to the western blotting protocol and the lysate was rotated at 4 °C for 45 min. Beads were washed with equilibration buffer twice. Western blot procedure was implemented as described above.

### Combination index analysis

Isobolograms were generated using Calcusyn software (Biosoft, Cambridge, UK). Combination index values were calculated according to the method of Chou and Talalay as described in the manual.^62^ For each drug, 72-h IC_50_ values were determined by CellQuantiBlue assay. Concentration-response curves were generated for each compound and combination using four 80% dilutions.

### RhoA overexpression

Human RhoA was amplified by PCR using the following primers designed to yield a C-terminal faneslyation sequence (-CVLS): 5′-GCTATCGAATTCATGGCTGCCATCCGGAAGA-3′ (forward) and 5′-GCTATCCTCGAGTCAGCTCAGCACGCAACCAGATTTTTTCTTCCCA-3′ (reverse). The amplicon was ligated into the pcDNA3.1(+) plasmid using the *Eco*RI and *Xho*I restriction sites. Before electroporation, Molt-4 cells were incubated overnight without antibiotics. Cells were then washed and resuspended in serum-free media at 2.5 × 10^7^ cells/ml. In all, 20 *μ*g of DNA was added to 400 *μ*l of cells for 10 min at RT. Cells were pulsed using the exponential protocol at 316 V and 500 *μ*F and allowed to incubate at RT for 15 min. Cells were allowed to rest for 20 h before selection with G418 at 400 *μ*g/ml.

### Statistical analysis

ANOVA (one-way) was used to calculate significance. Comparisons were done relative to the control or between pairs of conditions as indicated in the graphs. Columns in bar and line graphs represent the mean±S.D. of the indicated number of experimental replicates. An *α* level of 0.05 was used as the level of significance.

## Figures and Tables

**Figure 1:**
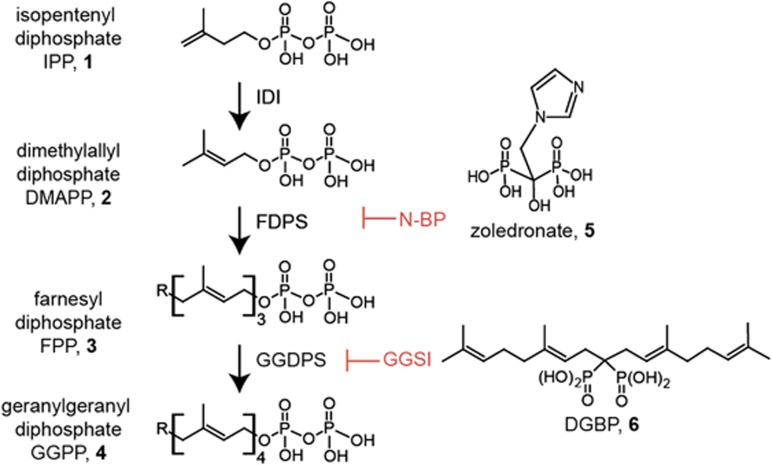
Biosynthesis of GGPP and known isoprenoid biosynthesis pathway inhibitors. Bisphosphonates such as zoledronate and DGBP inhibit isoprenoid biosynthesis by targeting the enzymes FDPS and GGDPS, respectively. Isopentenyl diphosphate isomerase (IDI) catalyzes the isomerization of isopentenyl diphosphate (1) into DMAPP (2). FDPS then takes one equivalent of DMAPP and two equivalents of isopentenyl diphosphate to form FPP (3) (R = H). This step can be inhibited by zoledronate (5). GGDPS then catalyzes the condensation of FPP and isopentenyl diphosphate to form GGPP (4) (R = H). This step can be inhibited by novel inhibitor DGBP, thus depleting levels of GGPP

**Table 1:** Selectivity of DGBP and zoledronate for malignant *versus* primary cells

	**IC_50_ (μM) (95% CI)**			**Selectivity *versus*PBMC**	
	**PBMC**	**Molt-4**	**Jurkat**	**Molt-4**	**Jurkat**
DGBP	1200 (480–3000)	15 (4.7–48)	30 (14–64)	80	40
Zoledronate	63 (32–120)	52 (37–74)	81 (46–140)	1.2	0.78
Fold difference	0.053	3.5	2.7		

Abbreviation: DGBP, digeranyl bisphosphonate
