# Supplementary material for: Geranylgeranyl diphosphate synthase inhibition induces apoptosis that is dependent upon GGPP depletion, ERK phosphorylation and caspase activation
Source: Cell Death Dis. 2017 Mar 16;8(3):e2678–. doi: 10.1038/cddis.2017.101 (PMC5386513; doi:10.1038/cddis.2017.101)
Supplement: Supplementary Information [file cddis2017101x1.docx]

| DGBP [μM] | Zoledronate [μM] | CI |
| --- | --- | --- |
| 41 | 41 | 7.5 |
| 51 | 51 | 6.9 |
| 64 | 64 | 4.8 |
| 80 | 80 | 3.9 |
| 100 | 100 | 4.4 |

**Table S1. Combination Index (CI) for experimental values of bisphosphonate combinations.**

**
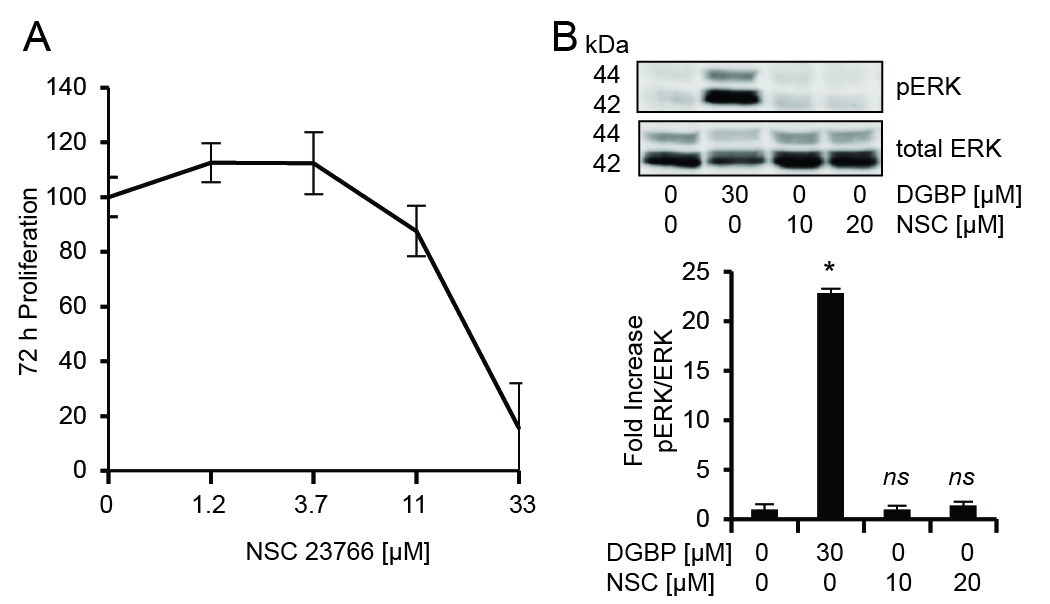
**

**Figure S1. Rac inhibitor decreases proliferation but does not alter ERK phosphorylation.** A) Dose response of Rac inhibitor, NSC 23766, on proliferation of Molt-4 cells. Data is representative of five independent experiments B) Western blot analysis of p-ERK levels with NSC 23766 treatment. Data represents mean ± SD, n=3. * indicates significant difference with respect to untreated control conditions.
